# Clinical features and management approaches for Urinary Incontinence in Older Adults: Evidence from Three Hospitals in Qatar

**DOI:** 10.3126/nje.v14i2.69365

**Published:** 2024-09-02

**Authors:** Asma Abbas, Brijesh Sathian, Mostafa Elawady, Shafi Hashmath Ulla Khan, Amir Ibrahim Abdalla, Ahmed Hayati Mohamed Ahmed Hasabelgawy, Ardalan Abdolgafor Ghafouri, Susan Mohieldeen Osman, Abdelrahman Hamad O Alzubier, Osama Elnour Abdelnour Idris, Hanadi Al Hamad

**Affiliations:** 1 Geriatrics and long-term care department, Rumailah Hospital, Doha, Qatar; 2 Hamad Medical Corporation, Qatar

**Keywords:** Urinary incontinence, older adults, Alphablockers, Anticholinergics, risk factors, Qatar

## Abstract

**Background:**

Urinary incontinence (UI) is a common medical problem that seriously affects patients' physical, psychological, social, and financial well-being. To identify the most effective drug management techniques, this retrospective study aimed to describe the demographics, etiology, and medical traits of people with UI.

**Methods:**

This retrospective study was conducted at Rumailah Hospital, Ambulatory Care Centre, and Hamad General Hospital to investigate UI risk factors, causes, and management practices in people aged ≥ 65 years within the Hamad Medical Corporation (HMC) in Qatar.

**Results:**

The 272 patients enrolled in the study had a marked male preponderance, and a larger percentage of non-Qatari residents than Qataris residents. Solifenacin (24.9%), Tolterodine (4.1%), and Oxybutynin (1.1%) were the most commonly administered anticholinergic drugs, while Tamsulosin (82.9%), Alfuzosin (14.5%), and Doxazosin (1.7%) were the most frequently prescribed alpha-blockers.

**Conclusion:**

This study underscores the importance of investigating UI in institutionalized older adult populations considering the limited research available in Qatar. The identified preventable risk factors can be proactively addressed to mitigate UI. This study also highlights the need for thorough documentation of the diagnosis and reasons for improving the standards of patient care. The findings of this study provide important information that can be used to design medication management methods for enhancing patient outcomes.

## Introduction

According to the International Continence Society (ICS), urinary incontinence (UI) is a complaint of unintentional urine loss [1]. This distressing medical condition is not a normal consequence of aging, and requires prompt medical intervention. The negative repercussions of UI extend beyond the physical realm and significantly impact patients' quality of life, giving rise to psychological, social, and economic challenges [2-3]. Hence, a holistic and multidisciplinary approach to UI management is highly recommended to address the multifaceted aspects of this condition [2-3].

UI encompasses three primary types: mixed, urge, and stress incontinence, each presenting unique challenges for the affected individuals. UI is a prevalent clinical disorder that affects individuals of diverse races and cultures worldwide, warranting thorough investigation and comprehensive management strategies [4-12].

Sex disparities exist in UI prevalence, with women being twice as likely to experience UI as men [13]. Notably, at least one-third of older women experience UI, indicating a significant burden on this demographic [13]. A study conducted in Qatar in 2012 revealed an UI prevalence of approximately 21% among women, which is comparable to the rates observed in Saudi Arabia [4]. Notably, UI prevalence in women across various studies ranges from 10% to 60% [4-12]. Likewise, in Europe, the prevalence of UI among female respondents varies by country, with estimates ranging from 23% to 44% [12]. Among men, the UI prevalence falls within the range of 7–16% [14-15].

Beyond its physical effects, UI is also closely related to mental health, particularly depression. In older persons, depressive symptoms have been linked to higher mortality rates, greater comorbidity, increased use of healthcare services, and decreased overall quality of life [16]. Depressive symptoms are more likely to affect older adults with chronic diseases [16]. Frequency, urgency, nocturia, and actual disease can all significantly hinder daily activities, self-esteem, and social contact, which can lead to anxiety, depressed symptoms, gossip, and social exclusion. Despite the clear interaction between UI and depression, few studies have examined this relationship, highlighting the need for further investigation and comprehension [16].

According to the Planning and Statistics Authority 2020 census report, Qatar's older adult population will include over 75068 people aged 60 years and over, and the prevalence of UI is expected to increase with this growth [17]. Therefore, it is crucial to conduct a thorough examination of the causes, risk factors, and comorbidities of UI in older people as well as to identify the best methods for managing their medications.

The main goals of our study were to outline the most efficient approaches to medication management while also providing a thorough assessment of the demographics, etiology, comorbidities, and risk factors of UI in the older adult population of Qatar. Our study aimed to enhance the quality of life of older adults with UI and provide insightful information that will help promote public health in UI management.

## Methodology

### Study design and participants

UI among people aged ≥ 65 years at the Hamad Medical Corporation (HMC), Qatar, was included in this retrospective study, which investigated its risk factors, causes, and treatment methods. Between November 2017 and November 2018, the study was conducted at Rumailah Hospital, Ambulatory Care Center, and Hamad General Hospital. All older adults who received UI drugs at any time during the study period were enrolled. No interventions were performed during the study period.

### Outcome measures

Comprehensive data regarding risk factors, causes, and management practices related to UI were collected for analysis. The variables collected included age, sex, nationality, depression, benign prostatic hyperplasia (BPH), history of recurrent UTI, urinary catheter use, medications, comorbidities, bacterial organisms, smoking habits, constipation, and obesity.

### Ethics statement

The study involving human participants was reviewed and approved by the Medical Research Centre of Hamad Medical Corporation (MRC-01-18-425). No identifiable image data statements have been presented in the manuscript.

### Statistical analysis

UI, constipation, benign prostatic hyperplasia (BPH), depression, dementia, obesity, and smoking in the study population were summarized using descriptive statistics. Means and standard deviations of continuous variables are shown, whereas percentages of categorical variables are used. Multivariate logistic regression analysis was performed to examine the relationship between UI and constipation, BPH, depression, dementia, obesity, and smoking. The associations were quantified using odds ratios (ORs) and 95% confidence intervals (CIs). Statistical significance was determined using a p-value threshold of 0.05. All statistical analysis were performed using the R-4.3.2 for Windows software.

## Results

This study included 272 patients with an average age of 74.9 years. There were more non-Qatari people in the patient population and more men than in women (Table 1). Constipation, benign prostatic hyperplasia (BPH), depression, and dementia had prevalence rates of 32.3%, 49%, 32%, and 6.2%, respectively. Furthermore, 9.6% of the patients smoked and 60% of the patients were obese.

Escherichia coli (E. coli) was the most frequently isolated bacteria in UTI cases (9.6%), followed by Klebsiella (6.2%), and Enterococcus faecalis (4.4%). Chronic kidney disease (CKD) was one of the most prevalent comorbidities in the study sample, affecting 37.6% of patients. A total of 64% of the patients had diabetes mellitus type 2 (DM2), while 21% had ischemic heart disease (IHD). Asthma (9.2%), Alzheimer's disease (1.8%), congestive heart failure (CHF) (4%), cerebrovascular accidents (CVA) (10.3%), and dementia (6.2%) were among the other comorbidities noted. Most patients had a history of taking alpha blockers, with tamsulosin being the most frequently prescribed medication (82.9%). Solifenacin was the most often prescribed anticholinergic drug (24.9%).

Constipation and anticholinergic medication use were shown to be significantly linked (p 0.05, Table 2). However, there was no statistically significant association between constipation and the other traits. Similarly, no statistically significant correlation was found between any other variables and recurrent UI; however, there was a significant correlation between recurrent UI and BPH (p 0.05, Table 3).

## Discussion

UI is a prevalent and serious health issue that affects people of all ages and has varying levels in different groups and circumstances [18-22]. Our study found that the prevalence of UI in older men ranged from 33.94 to 60.3%, whereas it ranged from 31.5 to 59.2% in older women. These findings are consistent with past studies conducted in various countries and emphasize the value of understanding risk factors and mitigation strategies for UI among older adults [21,22].

It is noteworthy that occurrence rates differed across settings in the community and among institutions. In institutional settings, prevalence rates are comparable for both sexes, in contrast to general communities, where females frequently experience higher rates of UI than males [23, 24]. This difference can be attributed to older men's higher prevalence of comorbidities, polypharmacy, and physical impairments, which may limit their mobility, access to food, and use of restrooms [25].

Given the increasing number of older adults in Qatar, it is essential to address UI as a severe health concern to enhance their general well-being and quality of life [26-28]. It is critical to reduce stigma and encourage self-care practices, because the stigma associated with UI can prevent people from receiving the care they need. UI indicators and symptoms that are more delicately stated during conversations with older people may also be beneficial [26]. It should be noted that long-term care facilities in Qatar need to improve their UI prevention measures, particularly in light of the higher risks of cognitive decline, depression, chronic illness, and falls among older persons living alone or in such facilities [28]. If UI is addressed as an important health concern in Qatar's older population, it will improve health outcomes and general well-being.

Consistent with earlier studies that showed a higher incidence of constipation in patients taking such medications [29, 30], our findings showed a substantial relationship between the use of anticholinergic medications and constipation. This finding emphasizes the significance of cautious anticholinergic medication prescriptions for elderly patients with constipation. Additionally, the substantial correlation between recurrent UI and benign prostatic hyperplasia (BPH) lends credence to the body of research supporting this relationship [31]. As a result, efficient treatment of recurrent UI requires careful management of BPH in elderly patients.

However, it is important to note that this study has several limitations. Owing to its retrospective design, it is difficult to demonstrate a causal relationship between variables, and the data were obtained only from three particular locations, which may limit its applicability to different environments. Future longitudinal investigations are therefore required to confirm these results and investigate additional potential risk factors for constipation and recurrent urinary tract infections (UTI) in older people.

## Conclusion

In conclusion, this study offers insightful information about the risk factors and approaches to UI management in older patients in Qatar. Healthcare professionals should consider the therapeutic importance of the discovered correlations between BPH and recurrent UI, anticholinergic medications, and constipation when treating elderly patients with these problems. Qatar can enhance preventive measures in long-term care institutions and address UI as an important health problem to enhance the health and well-being of the elderly population.

## Figures and Tables

**Table 1:** Socio-demographic and clinical characteristics

Variables	n (%)
**Age group**	
**60-69**	84 (31%)
**70-79**	123 (45.4%)
**80+**	64 (23.6%)
**Gender**	
**Male**	233 (85.9%)
**Female**	38 (14.0%)
**Nationality**	
**Qatari**	103 (37.9)
**Non-Qatari**	169 (62.1)
**Depression**	61 (22.4%)
**BPH**	134 (49.2%)
**History of recurrent UTI**	208 (80%)
**Urinary catheter**	233 (85.6%)
**Medications**	
**Anti-cholinergic**	
**Oxybutynin**	3 (1.1%)
**Solifenacin**	66 (24.9%)
**Tolterodine**	11 (4.1%)
**Alpha-blockers**	
**Tamsulosin**	195 (82.9%)
**Alfuzosin**	34 (14.5%)
**Doxazosin**	4 (1.7%)
**Co-morbidities**	
**CKD**	86 (37.6)
**Asthma**	25 (9.2%)
**Alzheimer’s disease**	5 (1.8%)
**DM2**	174 (64%)
**CHF**	11 (4%)
**IHD**	57 (21%)
**CVA**	28 (10.3%)
**Dementia**	7 (6.2%)
**CA hx**	7 (2.6%)
**Grand mal seizures**	1 (0.4%)
**Epilepsy**	1 (0.4%)
**Parkinson’s disease**	4 (1.5%)
**BPH**	134 (49.2%)
**Bacterial organism**	
**E. coli**	26 (9.6%)
**Pseudomonas**	7 (2.6%)
**Klebsiella**	17 (6.2%)
**Enterococcus fecalis**	12 (4.4%)
**Smoking:**	
**Former smoker**	31 (11.4%)
**Smoker**	26 (9.6%)
**Never smoker**	78 (28.7%)
**Constipation**	88 (32.3%)
**Obesity (n=58)**	35 (60.4%)

**Table 2:** Multivariate analysis of constipation and other variables

Variables	Constipation		AOR (95% CI)	P-value
	**No**	**Yes**		
**Age**	75+8.5	74.9+7.4	0.99 (0.96, 1.03)	0.8
**Gender**				
**Male**	159 (68.2)	74 (31.8)		
**Female**	24 (63.2)	14 (36.8)	0.86(0.36, 2.05)	0.7
**Anti-cholinergic**				
**No**	139(72.4)	53 (27.6)		
**Yes**	45(56.3)	35 (43.8)	2.1(1.15, 3.81)	0.02*
**Diabetes**				
**No**	66(67.4)	32 (32.7)		
**Yes**	118 (67.8)	56 (32.2)	0.89 (0.51, 1.54)	0.7
**CVA**				
**No**	168 (68.9)	76 (31.2)		
**Yes**	16 (57.1)	12 (42.9)	1.6(0.71, 3.65)	0.3
**BPH**				
**No**	93 (67.4)	45 (32.6)		
**Yes**	91 (67.9)	43 (32.1)	0.99 (0.56, 1.76)	0.9

*Statistically Significant

**Table 3:** Multivariate analysis of Recurrent UTI and other variables

Variables	Recurrent UTI		AOR (95% CI)	P-value
	**No**	**Yes**		
**Age**	76.9+7.4	74.4+8.3	0.96 (0.93, 0.99)	0.8
**Gender**				
**Male**	42 (18.6)	182 (81.3)		
**Female**	10 (27.8)	26 (72.2)	1.19(0.46, 3.1)	0.7
**Anti-cholinergic**				
**No**	33(18.1)	149 (81.8)		
**Yes**	19(24.4)	59 (75.6)	0.65(0.31, 1.35)	0.2
**Diabetes**				
**No**	14(15.1)	79 (84.9)		
**Yes**	38 (22.6)	129 (77.3)	0.58 (0.28, 1.2)	0.2
**CVA**				
**No**	46 (19.7)	187 (80.3)		
**Yes**	6 (22.2)	21 (77.8)	1.2(0.43, 3.3)	0.7
**CKD**				
**No**	31 (17.5)	146 (82.5)		
**Yes**	21 (25.3)	62 (74.7)	0.6 (0.3, 1.2)	0.2
**BPH**				
**No**	38(29)	93(71)		
**Yes**	14 (10.9)	115 (89.2)	3.45 (1.66, 7.17)	0.01*

*Statistically Significant
